# Fatal Outbreak in Tonkean Macaques Caused by Possibly Novel Orthopoxvirus, Italy, January 2015^1^

**DOI:** 10.3201/eid2312.162098

**Published:** 2017-12

**Authors:** Giusy Cardeti, Cesare Ernesto Maria Gruber, Claudia Eleni, Fabrizio Carletti, Concetta Castilletti, Giuseppe Manna, Francesca Rosone, Emanuela Giombini, Marina Selleri, Daniele Lapa, Vincenzo Puro, Antonino Di Caro, Raniero Lorenzetti, Maria Teresa Scicluna, Goffredo Grifoni, Annapaola Rizzoli, Valentina Tagliapietra, Lorenzo De Marco, Maria Rosaria Capobianchi, Gian Luca Autorino

**Affiliations:** Istituto Zooprofilattico Sperimentale del Lazio e della Toscana M. Aleandri, Rome, Italy (G. Cardeti, C. Eleni, G. Manna, F. Rosone, R. Lorenzetti, M.T. Scicluna, G. Grifoni, G.L. Autorino);; L, Spallanzani National Institute of Infectious Diseases, Rome (C.E.M. Gruber, F. Carletti, C. Castilletti, E. Giombini, M. Selleri, D. Lapa, V. Puro, A. Di Caro, M.R. Capobianchi);; Fondazione Edmund Mach di San Michele all’Adige, Trento, Italy (A. Rizzoli, V. Tagliapietra);; Parco Faunistico Piano dell'Abatino, Poggio San Lorenzo, Italy (L. De Marco)

**Keywords:** orthopoxvirus infection, diagnosis, epidemiology, Tonkean macaque, *Macaca tonkeana*, vaccination, cowpox virus, ectromelia virus, metagenomics, cluster analysis, phylogeny, Italy, viruses, zoonoses

## Abstract

In January 2015, during a 3-week period, 12 captive Tonkean macacques at a sanctuary in Italy died. An orthopoxvirus infection was suspected because of negative-staining electron microscopy results. The diagnosis was confirmed by histology, virus isolation, and molecular analysis performed on different organs from all animals. An epidemiologic investigation was unable to define the infection source in the surrounding area. Trapped rodents were negative by virologic testing, but specific IgG was detected in 27.27% of small rodents and 14.28% of rats. An attenuated live vaccine was administered to the susceptible monkey population, and no adverse reactions were observed; a detectable humoral immune response was induced in most of the vaccinated animals. We performed molecular characterization of the orthopoxvirus isolate by next-generation sequencing. According to the phylogenetic analysis of the 9 conserved genes, the virus could be part of a novel clade, lying between cowpox and ectromelia viruses.

Genus *Orthopoxvirus* virions are brick-shaped and replicate in the cytoplasm of eukaryotic cells (*1*). Some orthopoxviruses have limited host ranges; for example, ectromelia virus (ECTV) has only been described infecting captive colonies of laboratory mice (*2*,*3*). Other orthopoxviruses can infect multiple animal species; for example, cowpox virus (CPXV) has been observed on multiple occasions to spill over from its natural reservoir (presumably small wild rodents) to a wide variety of accidental hosts, including humans (*4*). The 3 orthopoxviruses raccoonpox, skunkpox, and volepox viruses are recognized as endemic in North America and are referred to as New World orthopoxviruses (*5*). The 6 orthopoxviruses variola virus, vaccinia virus (VACV), camelpox virus, monkeypox virus, ECTV, and CPXV are recognized as originating from the Eurasian continent and are referred to as Old World orthopoxviruses (*6*). Phylogenetic analyses have determined that CPXV is composed of multiple paraphyletic clades: VACV–like clade; variola virus–like clade; and CPXV clades 1, 2, and 3 (*4*,*7*). Moreover, analyses of 2 novel orthopoxviruses discovered in the country of Georgia (*8*) and the US state of Alaska (*9*) indicated they represented lineages distantly related from all previously known Old World and New World orthopoxviruses.

In Europe, western Russia, and northern and central Asia, CPXV is endemic, and in Europe, the numbers of reports are increasing (*10*). Many studies have explored the variable pathogenic potential of CPXV, observing that virulence and the clinical manifestations of a given strain are often correlated with the affected host species (*4*). In particular, exotic animals from zoos and circuses are reported to be highly susceptible to CPXV infections (*11*–*15*).

Among nonhuman primates (NHPs), orthopoxvirus infections have been reported in New World monkeys (*16*), Barbary macaques (*13*), squirrel monkeys (*17*), and tamarins (*14*), but these infections have not been described in the Tonkean macaque (*Macaca tonkeana*). The Tonkean macaque, belonging to the *Cercopithecidae* family, is found in 4 protected areas in central Sulawesi (*18*); a few social groups live in 4 rescue centers in Europe, including in Italy, and in central America. Because of the limited number of these animals in sanctuaries, they are infrequently observed infected with orthopoxviruses, and thus, little information has been published regarding their disease signs and symptoms.

A few cases of orthopoxvirus infection have been reported in Italy. In southern Italy, orthopoxvirus infections with CPXV have been described in domestic ruminants (*19*). At a farm in the region of Lazio, Italy, a CPXV outbreak occurred involving 7 llamas, which were suspected to have been infected via infected mice that were introduced to feed the birds of prey at the farm (*20*). Zoonotic infections caused by 2 almost identical orthopoxvirus isolates occurred in 2005 and 2007 in 2 veterinarians from northeastern Italy who acquired the infections during 2 separate incidents from 2 different infected cats (*21*). Although definitive taxonomic assignment of these orthopoxviruses was not conclusive, hemagglutinin (HA) and *crmB* sequence analyses suggested possible segregation of these virus isolates from other previously described orthopoxvirus strains.

We describe a severe orthopoxvirus epidemic that occurred in 2015 in a social group of Tonkean macaques hosted in a sanctuary for wild and exotic animals in a wooded area in central Italy, where 146 NHPs and 240 other wild and domestic mammals were maintained. To prevent other cases in NHPs, we undertook an immunization protocol and conducted epidemiologic investigations to detect orthopoxvirus carriers. We also performed taxonomic characterization to determine its relatedness to other orthopoxviruses.

## Methods

The colony of Tonkean macaques was introduced to the sanctuary in 2007 from the Strasbourg Primate Centre of Strasbourg University (Strasbourg, France); the original stock had been imported into France from Indonesia in 1972. The colony hosted in the center at the time of the outbreak comprised 54 animals housed in 4 wide enclosures separated from each other at different distances.

**Figure 1:**
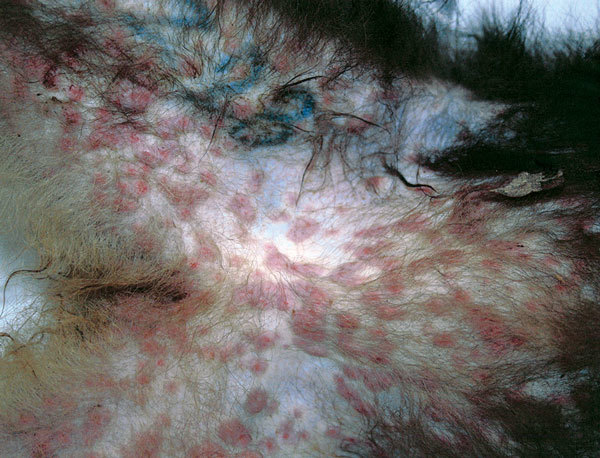
Crater-shaped skin lesions at inguinal region of Tonkean macaque (*Macaca tonkeana*) housed at animal sanctuary, Italy, January 2015.

In January 2015, twelve of the 18 Tonkean macaques housed in an enclosure located in the northern part of the natural reserve died within a 3-week period. The social group housed in the affected enclosure included both male and female animals, 1–20 years of age. Two macaques died within 48 hours after disease onset with severe respiratory syndrome. In the following weeks, 13 animals from the same enclosure displayed signs of depression, nausea, respiratory distress, and neurologic disease, and in several animals, skin and mucosa lesions developed (Figure 1). Ten of these 13 macaques died within 15 days after the appearance of signs and symptoms; 2 of 13 recovered after 6 and 8 days; and the remaining animal, an adult male, recovered but experienced long-lasting effects from the infection. This macaque had severe lesions limited to the right side of the face and the eye. In April, when this animal underwent surgery for eye ablation, oral and rectal swabs and a peripheral blood sample were acquired to detect virus and perform serologic studies.

The remaining 3 macaques in the same enclosure were constantly symptomless. Three days after the beginning of the outbreak, 2 of these macaques were immediately isolated in a separate area, and the third, a 1-year-old, was left with the mother, which recovered from the illness. None of the other 9 NHP species, wild ruminants, wild boars, donkeys, raccoons, cats, or dogs hosted in the sanctuary showed any clinical signs related to orthopoxvirus infection in the following 12-month observation period.

At postmortem examination, major organs and skin lesions were collected from all dead Tonkean macaques and processed for routine histologic (hematoxylin and eosin) staining. Virus detection by negative-staining electron microscopy (EM) was conducted with skin lesion samples from 10 animals (*22*). We extracted nucleic acid from homogenates of skin and lungs taken from 4 of the macaques and performed a molecular diagnostics investigation by using orthopoxvirus-specific PCR assays. We performed an orthopoxvirus-specific SYBR Green (ThermoFisher Scientific, Waltham, MA, USA) real-time PCR targeting *crmB* (*23*), and to confirm the first result, we tested all samples with an additional endpoint PCR targeting the orthopoxvirus HA gene. We tested 1 tissue sample for the genes encoding acidophilic-type inclusion body (ATI) protein, chemokine-binding protein (K2R), 602-kDa protein, and 14-kDa fusion protein (A27L).

Using tissue homogenates of the skin and tongue mucosa, brain, lungs, liver, spleen, heart, mesenteric lymph nodes, and intestines of the 12 dead animals (a total of 70 samples), we conducted an additional SYBR Green real-time PCR to confirm the presence of orthopoxvirus in all affected animals (Table 1). We performed virus culture by taking tissue homogenates from up to 3 dead animals and inoculating them on Vero cells (CCL-81; American Tissue Culture Collection, Manassas, VA, USA). We sequenced the whole genome of the virus isolate by using the metagenomic approach with the Ion Torrent Personal Genome Machine platform for next general sequencing (ThermoFisher Scientific, Waltham, MA, USA). In brief, we grew the virus to passage 4 (titer 1 × 10^6.9^ 50% tissue culture infectious dose/mL) and concentrated it by ultracentrifugation. Then, we extracted pellet-associated DNA with E-Gel SizeSelect Agarose Gels (ThermoFisher Scientific) and quantified with Qubit dsDNA HS Assay Kit (ThermoFisher Scientific). We prepared DNA libraries with the Ion Xpress Plus gDNA Fragment Library Kit (ThermoFisher Scientific). We filtered sequence reads with the VirFind tool (http://virfind.org/j/) and de novo assembled sequences with Newbler version 2.5.3 (454 Life Sciences, Branford, CT, USA). We aligned the 5 major contigs (length 13,743–108,913 nt) to CPXV-Germany1998–2 (GenBank accession no. HQ420897.1) with MAUVE software (*24*) and concatenated the sequences. Inverted terminal repeats were excluded from the genome reconstruction.

**Table 1:** Virologic examination of samples collected from 12 dead Tonkean macaques, Italy, January 2015*

Method	Skin	Tongue mucosa	Brain	Lungs	Liver	Spleen	Myocardium	Lymph node	Intestine	Total
Negative-staining EM	10/10	1/1	NA	NA	NA	NA	NA	NA	0/12	11/23
SYBR Green real-time PCR	10/10	1/1	3/3	12/12	10/12	12/12	3/3	5/5	3/12	59/70
Cell culture	3/3	1/1	3/3	3/3	2/2	2/2	3/3	NA	0/3	17/20

*Values are no. positive/no. total. EM, electron microscopy; NA, not analyzed.

We conducted epidemiologic investigations to examine virus transmission between working personnel and other animal species maintained in the reserve and to identify possible orthopoxvirus carriers (*13*). During February 2015–April 2016, the following animals in the same sanctuary died without specific symptoms: 9 NHPs, 2 foxes, and 1 cat. We conducted a virologic investigation for orthopoxvirus infection with the lungs, livers, spleens, and intestines of these dead animals.

After the outbreak, we initiated a 1-year rodent control program inside and in proximity to the enclosures. We captured 11 live mice (4 *Apodemus flavicollis* and 7 *Microtus* spp.) and 93 dead wild gray rats (*Rattus rattus*) by using multicapture live traps (Ugglan Special Mouse Trap 2; Grahnab, Hillerstorp, Sweden) (*25*) and electromechanical equipment. Lungs, liver, spleen, and small intestines of these animals were stored at –80°C until virologic analysis.

**Figure 2:**
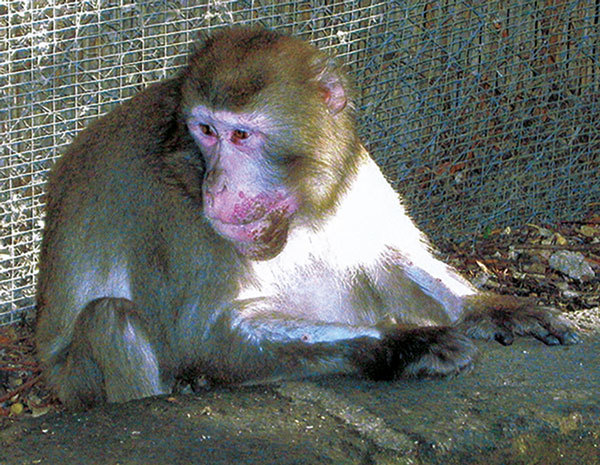
Crater-shaped skin lesions on face of Japanese macaque (*Macaca fuscata*), Italy, 2003.

The sanctuary owner reported that 1 Japanese macaque (*M. fuscata*) had similar skin lesions (Figure 2) in 2003, and at that time, infection with a herpes zoster virus had been ruled out. This animal was still alive and was sampled for serologic investigations at the time of the 2015 outbreak.

We performed a serologic investigation with blood samples (stored at −20°C after collection) taken from 30 various mammals (Table 2) and 62 NHPs (Table 3) housed at the sanctuary, as well as 11 mice and 56 dead rats. Detection of orthopoxvirus antibodies from NHPs and small rodents was performed by indirect immunofluorescence antibody (IFA) assay with homemade slides that were seeded with Vero-E6 cells and infected with the smallpox vaccine virus Lancy-Vaxina (*21*). To detect orthopoxvirus-specific antibodies in other mammals, we used a virus neutralization assay (*20*). We also conducted an epidemiologic and serologic investigation with the sanctuary staff (N = 11; group included veterinarians, researchers, and maintenance personnel) by performing a site visit and interviews and collecting blood samples.

**Table 2:** Serologic analysis of serum samples collected from various mammal species housed at animal sanctuary, Italy, January 2015

Species	Virus neutralization test, no. positive/no. total	Antibody titer*
Wolf	0/1	Negative
Llama	0/8	Negative
Roe deer	0/1	Negative
Mouflon	0/1	Negative
Goat	0/4	Negative
Badger	0/1	Negative
Donkey	0/2	Negative
Cat	0/12	Negative
Total	0/30	

*Threshold dilution was 1:4.

**Table 3:** Serologic analysis of serum samples from nonhuman primate species housed at animal sanctuary, Italy, January 2015*

Species	IFA IgM test, no. positive/no. total (%)	IgM titer†	IFA IgG test, no. positive/no. total (%)	IgG titer,† range
Tonkean macaque (*Macaca tonkeana*)	0/30	Negative	8/30	1:40−1: 640
Cynomolgus macaque (*M. fascicularis*)	0/11	Negative	3/11	1:20−1:160
Barbary macaque (*M. sylvanus*)	2/12	1:20	2/12	1:20
Rhesus macaque (*M. mulatta*)	0/1	Negative	1/1	1:80
Japanese macaque (*M. fuscata*)	0/2	Negative	2/2	1:80
Tufted capuchin (*Sapajus apella*)	0/4	Negative	2/4	1:20
Grivet (*Cercopithecus aethiops*)	0/1	Negative	0/1	Negative
Hamadryas baboon (*Papio hamadryas*)	0/1	Negative	0/1	Negative
Total	2/62 (3.22)		18/62 (29.03)	

*IFA, immunofluorescence antibody. †Threshold dilution was 1:20.

## Results

**Figure 3:**
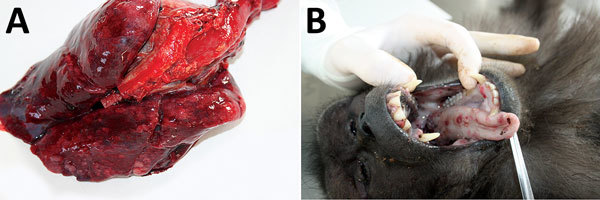
Results from necropsy of Tonkean macaque (*Macaca tonkeana*) from animal sanctuary, Italy, January 2015, showing severe congestion in the lungs (A) and erythematous papules and pustular lesions on the buccal and tongue mucosae (B).

**Figure 4:**
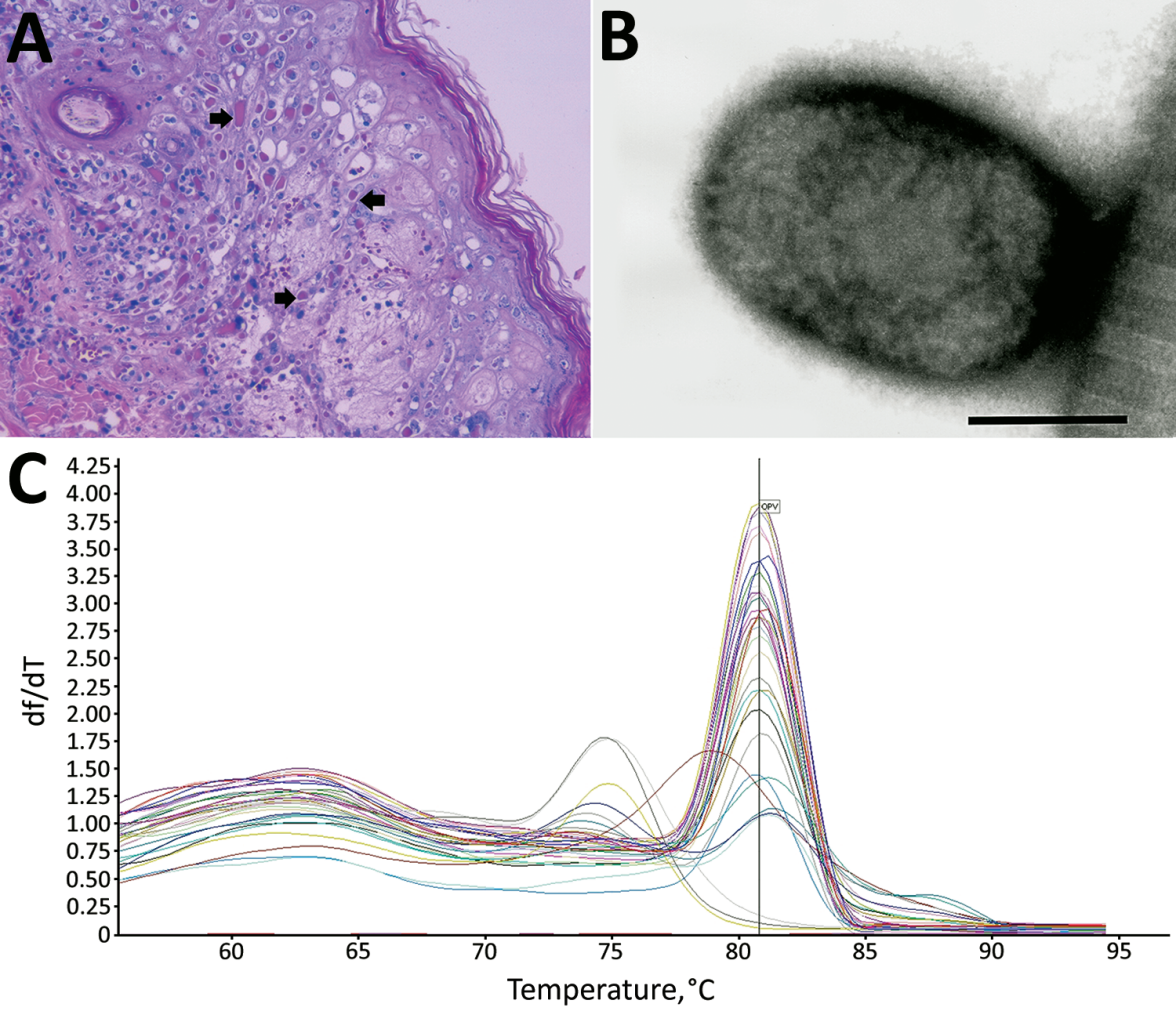
Results from necropsy of Tonkean macaque (*Macaca tonkeana*) from animal sanctuary, Italy, January 2015. A) Hematoxylin and eosin stain of cutaneous lesion. Focal epidermal necrosis, acanthosis ballooning degeneration, and acantholysis of keratinocytes was observed. Staining shows early vesiculation with eosinophilic intracytoplasmic inclusion bodies (arrows) in enlarged degenerated cells. B) Electron micrograph of skin lesion sample showing negatively stained brick-shaped viral particle of ≈160–220 nm, consistent with orthopoxvirus. Scale bar = 100 nm. C) SYBR Green (ThermoFisher Scientific, Waltham, MA, USA) real-time PCR melting curve of all tested samples. The *y*-axis shows the ratio of the change in fluorescence over the change in temperature. The average melting temperature (80.8°C ± 1°C) was consistent with that for the orthopoxvirus genome.

At necropsy, the 2 animals that died within 48 hours after symptom onset showed severe lung congestion (Figure 3, panel A) and hepatosplenomegaly. The 10 animals that died 5–15 days after onset of clinical symptoms had erythematous papular and pustular lesions on the face, in the oral cavity, on the tongue mucosa (Figure 3, panel B), and at the inguinal region. All cutaneous lesions were characterized by focal epidermal necrosis and early vesiculation with eosinophilic intracytoplasmic inclusion bodies in enlarged degenerated cells (Figure 4, panel A). The liver showed scattered foci of necrosis and moderate steatosis. Foci of necrosis at the lymphoid follicles and histiocytosis associated with hemorrhages were observed in the spleen and at the lymph nodes. In some cases, mild interstitial pneumonia was associated with focal necrosis of bronchial epithelium.

Negative-staining EM revealed the presence of brick-shaped particles morphologically consistent with orthopoxvirus in the skin lesions of 10 dead macaques (Figure 4, panel B). Vero cell cultures inoculated with skin lesion materials from infected monkeys showed the previously described cytopathic effect (*20*) 3 days after inoculation. A viable transmissible agent, which we named orthopoxvirus Abatino, was consistently isolated and confirmed to be an orthopoxvirus by negative-staining EM.

Confirming the negative-staining EM results, SYBR Green real-time PCR detected an orthopoxvirus genome in all organs from the 12 dead macaques (Table 1); the melting temperature of the amplicons (80.8°C ± 1°C; Figure 4, panel C) was identical for all tested samples and consistent with that of orthopoxvirus genomes (*23*). The presence of the HA sequence in tissues confirmed infection with an orthopoxvirus and ruled out the presence of monkeypox.

Oral and rectal swabs collected 3 months after the epidemic from the recovered male macaque were negative by all virologic analyses. Retrospective examination of the sanctuary records of the past 2 years excluded the possibility of introduction of this orthopoxvirus through contacts with other mammal species maintained in captivity.

All tissue samples from the 104 small rodents trapped during February 2015–June 2016 were negative for orthopoxvirus by PCR. The animals that died in the sanctuary after the epidemic were negative for orthopoxvirus by all virologic tests performed.

The Japanese macaque that had orthopoxvirus-like skin lesions in 2003 showed an orthopoxvirus-specific IgG (but not IgM) titer of 1:80 by IFA assay. The IFA assay showed orthopoxvirus-specific IgG in 8 (14.28%) of 56 rats and in 3 (27.27%) *Apodemus flavicollis* mice of 11 small rodents (mice and voles). Orthopoxvirus-specific IgM was never detected (Table 4).

**Table 4:** Immunofluorescence antibody testing of serum samples from small wild rodents trapped at animal sanctuary, Italy, January 2015*

	IgM			IgG	
Species	No. positive/no. total (%)	Titer*		No. positive/no. total (%)	Titer*
Gray rat (*Rattus rattus*)	0/56 (0)	Negative		1/56	1:20
				4/56	1:40
				3/56	1:80
Total	0/56 (0)			8/56 (14.28)	
Voles (*Microtus* spp.)	0/7	Negative		0/7	Negative
Yellow-necked mouse (*Apodemus flavicollis*)	0/4	Negative		3/4	1:40
Total	0/11 (0)			3/11 (27.27)	

*Threshold dilution was 1:20.

Among the NHPs outside of the affected enclosure, we detected orthopoxvirus IgG in 18 NHPs, 6 of which were asymptomatic Tonkean macaques maintained in an enclosure near the affected one. Orthopoxvirus IgM was detected at low titer only in 2 Barbary macaques that never showed clinical signs of orthopoxvirus infection (Table 3).

## Animal Vaccination

To prevent further infections, 96 NHPs of 8 species (*M. tonkeana*, *M. fascicularis*, *M. sylvanus*, *M. fuscata*, *M. mulatta*, *Sapajus apella*, *Chlorocebus aethiops*, and *Papio hamadryas*) were vaccinated during October–December 2015. Each animal received 2 doses of modified vaccinia virus Ankara (MVA) vaccine (Bavarian Nordic, Kvistgaard, Denmark) given 1 month apart, according to the producer’s immunization protocol. To assess possible vaccine-related adverse reactions, we monitored the NHPs for up to 10 days after dose administration and did not observe lesions at the vaccine inoculation site or general symptoms.

The immune responses to the vaccine were evaluated in a group of 10 animals (4 *M. tonkeana*, 5 *M. fascicularis*, and 1 *M. sylvanus*). Seven animals were negative for antibodies before vaccination, and 3 had a barely detectable baseline IgG titer. After vaccination, all monkeys showed a 2–5-fold increase of IgG titer. IgM were observed in the serum of 4 animals at or near the minimum threshold dilution (1:20) on the day of vaccine booster administration (Table 5). We did not detect viable virus or viral DNA shedding.

**Table 5:** Immunofluorescence antibody testing of vaccinated nonhuman primates after outbreak at animal sanctuary, Italy, January 2015*

Species	No. animals	IgM, T0/T30/T90	IgG, T0/T30/T90	No. (%) with IgG increase
Tonkean macaque (*Macaca tonkeana*)	4	Neg/1:20/neg	Neg/1:20/1:320	4 (100)
		Neg/neg/neg	Neg/1:20/1:80	
		Neg/neg/neg	Neg/neg/1:80	
		Neg/neg/neg	Neg/neg/1:40	
Cynomolgus macaque (*M. fascicularis*)	5	Neg/1:20/1:20	1:20/1:320/1:320	5 (100)
		Neg/1:80/1:80	1:20/1:80/1:80	
		Neg/neg/neg	Neg/1:20/1:80	
		Neg/1:20/1:20	Neg/1:20/1:80	
		Neg/neg/neg	Neg/1:80/1:160	
Barbary macaque (*M. sylvanus*)	1	Neg/neg/neg	1:20/1:20/1:80	1 (100)

*Threshold dilution for antibody titers was 1:20. Neg, negative; T0, first immunization; T30, booster dose administration; T90, blood collection 60 d after T30.

## Molecular Characterization of Virus Isolate

After the positive results of the first diagnostic molecular analysis, we performed additional genus-specific PCRs targeting HA, ATI, K2R, 602 kDa, and A27L with samples to better characterize the virus (*26*,*27*). We compared the complete nucleotide sequences of most amplicons with all orthopoxvirus full-genome sequences available in GenBank by using blastx+ version 2.2.28 (http://www.ncbi.nlm.nih.gov/books/NBK279690). The HA gene sequence was closely related to that of CPXV-Germany_1998_2 (GenBank accession no. HQ420897), with a 96% identity. K2R and 602-kDa protein gene sequences were closely related to those of ECTV-Moscow (GenBank accession no. AF012825), with a 96% identity for K2R (96% of gene sequence compared) and a 99% identity for 602-kDa protein. The A27L gene was closely related to those of ECTV-Naval and CPXV-Finland, with a 98% identity with both strains. Finally, the ATI gene sequence was most closely related to that of the cowpox virus CPXV-Norway1994-MAN (GenBank no. HQ420899.1), with a 98% identity.

Because of the discordant identity scores obtained with the preliminary molecular analyses, we sequenced the whole genome of the virus isolate by using next-generation sequencing. The sequence obtained was 202,990-nt long with a median coverage of 507 (range 28–817) nt. We identified 10 complete coding sequences and corrected insertions and deletions manually.

The first coding sequence identified was the HA gene, which had a median coverage of 485 (range 255–586) nt (GenBank accession no. KY100116). We used this gene to check the sequence identity with PCR results and to compare the homology with all available orthopoxvirus strains. Orthopoxvirus Abatino shared 99% nucleotide identity and 98% amino acid identity with ECTV-Moscow and 96% nucleotide identity and 94% amino acid identity with CPXV-Germany1998–2. Furthermore, we observed that Abatino had HA identity scores of 97% for the nucleotide sequence and 96% for the amino acid sequence with FelinePoxITA1 (GenBank accession no. EF612709.1), an orthopoxvirus isolate previously obtained in 2007 from a zoonotic case in northeastern Italy that does not have a definitive taxonomic assignment (*21*).

Because phylogenetic relationships based on HA are not considered reliable for assigning orthopoxvirus taxonomic relationships (*7*), we performed a more detailed analysis. Following a previously established pipeline (*28*,*29*), we performed core-genome selection, identity clustering, and phylogenetic reconstruction. Using blastx+, we identified the coding sequences of 9 conserved poxvirus genes (D1R, E6R, D5R, E9L, A7L, A10L, J6R, H4L, and A24R; VACV strain Copenhagen nomenclature; GenBank accession nos. KY100107–KY100115) uniformly distributed along the central region of the genome. The median coverage of the selected genes was 528 (range 80–743) nt. We aligned the concatenated sequences with homologous genes from available whole-sequence orthopoxvirus strains (*4*–*7*,*9*) by using MUSCLE version 3.8.31 (*30*).

**Figure 5:**
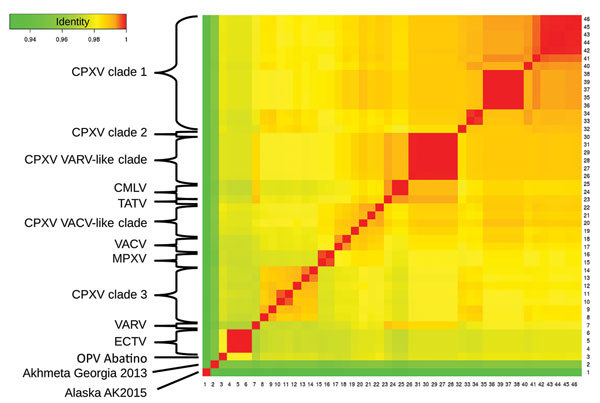
Identity between OPV Abatino, obtained from skin lesion of Tonkean macaque during outbreak at animal sanctuary, Italy, January 2015, and available OPV genomes on the basis of 9 concatenated conserved genes: A7L, A10L, A24R, D1R, D5R, H4L, E6R, E9L, and J6R. Red indicates more similarity, green less similarity. Sequences shown (GenBank accession nos.): 1) OPV Tena Dona AK2015 (KX914668–76); 2) OPV GCP2013 Akhmeta (KM046934–42); 4–6) ECTV-Moscow (AF012825.2), ECTV-Naval (KJ563295.1), ECTV-VR-1431 (JQ410350.1); 7) VARV-Bangladesh-1975 (L22579.1); 8–14) CPXV-HumLue09–1 (KC813494.1), CPXV-Germany1990–2 (HQ420896.1), CPXV-Francy2001-Nancy (HQ420894.1), CPXV-MarLei07–1 (KC813499.1), CPXV-Norway1994-MAN (HQ420899.1), CPXV-UK2000-K2984 (HQ420900.1), CPXV-BrightonRed (AF482758.2); 15–16) MPXV-Congo2003–358 (DQ011154.1), MPXV-Liberia-1970–184 (DQ011156.1); 17–18) VACV-IOC-B141 (KT184690.1), VACV-Lister (KX061501.1); 19–22) CPXV-Austria1999 (HQ407377.1), CPXV-HumLit08–1 (KC813493.1), CPXV-GRI90 (X94355.2), CPXV-Finland2000 (HQ420893.1); 23) TATV-Dahomey-1968 (DQ437594.1); 24–25) CMLV-0408151v (KP768318.1), CMLV-M96 (AF438165.1); 26‒31) CPXV-HumGra07–1 (KC813510.1), CPXV-RatKre08–2 (KC813505.1), CPXV-RatGer09–1 (KC813503.1), CPXV-RatAac09–1 (KC813501.1), CPXV-HumAac09–1 (KC813508.1), CPXV-HumKre08–1 (KC813512.1); 32) CPXV-Germany1998–2 (HQ420897.1); and 33–46) CPXV-Germany1980-EP4 (HQ420895.1), CPXV-HumPad07–1 (KC813496.1), CPXV-HumLan08–1 (KC813492.1), CPXV-RatHei09–1 (KC813504.1), CPXV-MonKre08–4 (KC813500.1), CPXV-JagKre08–1 (KC813497.1), CPXV-JagKre08–2 (KC813498.1), CPXV-Germany2002-MKY (HQ420898.1), CPXV-HumGri07–1 (KC813511.1), CPXV-HumMag07–1 (KC813495.1), CPXV-CatBer07–1 (KC813502.1), CPXV-HumBer07–1 (KC813509.1), CPXV-CatPox07–1 (KC813506.1), CPXV-BeaBer04–1 (KC813491.1). CMLV, camelpox virus; CPXV, cowpox virus; ECTV, ectromelia virus; MPXV, monkeypox virus; OPV, orthopoxvirus; TATV, taterapox virus; VACV, vaccinia virus; VARV, variola virus.

The identity score matrix (Figure 5) identified the 11 orthopoxvirus clusters described in previous reports (*4*,*9*). The identity score of orthopoxvirus Abatino with other orthopoxviruses ranged 93.26%–98.16%; the viruses with the highest identity scores to Abatino (98.14%, 98.15%, and 98.16%) were the 3 available ECTV sequences.

**Figure 6:**
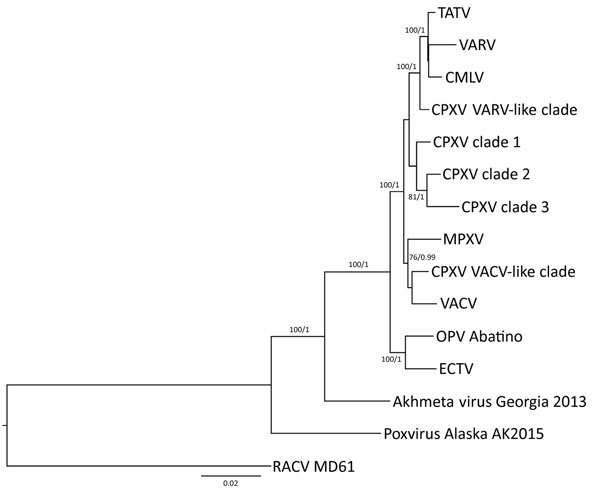
Phylogenetic analysis of OPV Abatino obtained from skin lesion of Tonkean macaque during outbreak at animal sanctuary, Italy, January 2015. Nine conserved genes (GenBank accession nos. KY100107–KY100115) obtained with next-generation sequencing were concatenated and aligned with the homologous concatenated sequences from representative OPV strains (GenBank accession no.): TATV-Dahomey-1968 (DQ437594.1), VARV-Bangladesh-1975 (L22579.1), CMLV-M96 (AF438165.1), CPXV-HumAac09–1 (KC813508.1), CPXV-Germany2002-MKY (HQ420898.1), CPXV-Germany1998–2 (HQ420897.1), CPXV-MarLei07–1 (KC813499.1), MPXV-Congo2003–358 (DQ011154.1), CPXV-Finland2000 (HQ420893.1), VACV-Lister (KX061501.1), ECTV-Moscow (AF012825.2), OPV GCP2013 Akhmeta (KM046934–42), and OPV Tena Dona AK2015 (KX914668–76). New World strain RACV-MD19 (GenBank accession no. FJ807746–54) was added to the analysis as an outgroup. We generated multiple alignments with MUSCLE version 3.8.31 (*30*) and built the phylogenetic tree by using the Bayesian Markov chain Monte Carlo model with MRBAYES version 3.2.5 (*31*) using the general time-reversible plus gamma model with 1 million generations, retaining a minimum of 10,000 posterior probabilities, and maximum-likelihood model RaxML version 8.1.24 (*32*) using the general time-reversible plus gamma with 1,000 pseudoreplicates. Numbers represent the reliability of the nodes with the minimum probability of 75% and minimum bootstrap value of 75. Scale bar indicates nucleotide substitutions per site. CMLV, camelpox virus; CPXV, cowpox virus; ECTV, ectromelia virus; MPXV, monkeypox virus; OPV, orthopoxvirus; RACV, raccoonpox virus; TATV, taterapox virus; VACV, vaccinia virus; VARV, variola virus.

Using the Bayesian Markov chain Monte Carlo model (*31*) and the maximum-likelihood model (*32*), we performed a phylogenetic analysis including a representative genome for each orthopoxvirus clade (Figure 6). The position of orthopoxvirus Abatino was consistent with the results of the identity heat map.

## Discussion

This epidemic indicated that Tonkean macaques are highly susceptible to infection with the isolated orthopoxvirus strain and might develop severe, fatal disease. Pathologic findings observed in dead animals were similar to those described in New World monkeys (*16*). Viable virus and viral DNA were detected not only in the cutaneous tissue and oral mucosa but also in many different organs, such as the liver and heart. Unfortunately, we could not detect viable virus in the intestinal tissues, and therefore, we cannot hypothesize regarding the possibility of viral transmission through feces among living animals.

The severe disease was limited to a single Tonkean macaque social group, and the results of SYBR Green PCR suggested the involvement of the same viral agent, even if not all isolates were genetically characterized. The high IgG titers in 6 macaques from the neighboring enclosure, however, indicated they became infected during the outbreak. Orthopoxvirus IgM and IgG detected in some NHPs in other enclosures support the hypothesis that the virus circulated in the natural reserve, but no orthopoxvirus symptoms were observed in these animals, and no viral DNA or viable virus was isolated from them. Also, no other mammals hosted in the sanctuary appeared to have been exposed to the virus.

The illness observed years before in the Japanese macaque that had orthopoxvirus-specific IgG suggests the possibility that an orthopoxvirus could have been circulating in the area since at least 2003, although no evidence was available to indicate whether that remote episode was caused by the same virus strain identified in the 2015 outbreak. Serologic evidence of orthopoxvirus infection in mice and rats confirms that mice and rats are susceptible to orthopoxviruses but does not definitively prove that they were infected with the virus responsible for the outbreak or establish their role as reservoirs, as has been described for rodents with other orthopoxvirus strains in previous studies (*10*,*13*).

The preliminary characterization of the isolated orthopoxvirus strain that was based on HA similarity analysis suggested that orthopoxvirus Abatino might be related to ECTV, CPXV clade 3, and a previous orthopoxvirus isolate that caused 2 zoonotic infections in northeastern Italy in 2005 and 2007 (*21*). On the other hand, the extended molecular characterization that was based on 9 conserved orthopoxvirus genes suggested that orthopoxvirus Abatino is in a distinct position with respect to all 11 orthopoxvirus clades, being more related to ECTV than to all other orthopoxvirus clades. ECTV occasionally infects laboratory mice populations (*2*) and is suspected to naturally spread among wild rodents, although ECTV has never been isolated from these animals (*3*). Host range differences between all previously described ECTVs and orthopoxvirus Abatino strongly suggest that Abatino does not belong to the ECTV lineage. We hypothesize that orthopoxvirus Abatino might be part of a novel, paraphyletic ECTV-like clade. A recombination event that affected the host range could also be considered as a possible origin for this virus, but a more extensive characterization is necessary.

MVA vaccine follow-up showed a detectable humoral immune response. Data on the antibody classes elicited by live-modified VACV administration in humans and NHPs are scarce or unavailable (*33*). However, the observations of Silva-Fernandes et al. (*34*) describing outbreaks in humans together with our data in vaccinated monkeys suggest that orthopoxvirus infections induce a limited IgM response. On the other hand, the low antibody titers detected could have been caused by a poor sensitivity of the IFA test adopted. Finally, the MVA vaccine was safe in the monkey populations vaccinated; we observed no adverse reactions.

The serologic surveillance conducted among sanctuary staff workers showed 1 staff member who did not previously receive orthopoxvirus vaccine and had close contacts with the affected Tonkean macaque group seroconverted without clinical signs, suggestive of an asymptomatic infection. However, alternative explanations for this seroconversion (e.g., previous exposure to a closely related virus) could not be ruled out.

This study might be considered alarming because orthopoxvirus vaccination has been discontinued globally since the late 1970s, which has resulted in the reduction of protective immunity over time not only against smallpox virus but also against a variety of other orthopoxviruses, raising the chances of orthopoxvirus infections occurring in humans. Orthopoxvirus infection in humans is not a notifiable disease in Italy, and because virologic diagnosis relies on specialized laboratories, orthopoxvirus infection is often not included among the differential diagnosis. Increased public awareness and linkage between human and veterinary health authorities is necessary to improve public health measures for the control of zoonotic orthopoxviruses.
